# Comparative investigation of the effects of conventional and skeletal anchorage pendulum appliances on dentofacial structures

**DOI:** 10.1097/MD.0000000000044512

**Published:** 2025-09-26

**Authors:** Ayca Aksoy, Oyku Ozen, Murat Kaan Erdem

**Affiliations:** a Private Practice, Ankara, Turkey; b Department of Oral and Maxillofacial Surgery, Lokman Hekim University, Faculty of Dentistry, Ankara, Turkey.

**Keywords:** Class II malocclusion, miniscrew, molar distalization, orthodontics, pendulum appliance, skeletal anchorage

## Abstract

**Background:**

Maxillary molar distalization is a common treatment approach for Class II malocclusions. This study aimed to compare the effectiveness and side effects of conventional pendulum appliances versus single and double miniscrew-supported pendulum appliances on skeletal and dentoalveolar structures.

**Methods:**

Thirty patients with skeletal Class I and dental Class II malocclusion were randomly allocated to 3 equal groups (n = 10 each): conventional pendulum appliance (Group 1), single miniscrew-supported pendulum (Group 2), and double miniscrew-supported pendulum (Group 3). Mean ages were 13.6 ± 1.2, 14.5 ± 1.1, and 14.3 ± 1.2 years, respectively. Treatment continued until bilateral Class I molar relationships were achieved. Pre- and post-distalization lateral cephalometric radiographs were analyzed for skeletal and dental changes.

**Results:**

The mean distalization period was significantly shorter in Group 3 (6.4 ± 0.5 months) compared to Group 2 (7.3 ± 0.5 months) and Group 1 (8.0 ± 0.7 months) (*P* < .05). All groups exhibited distal molar tipping with upper first molar long-axis angle to maxillary horizontal reference plane (°) increases of 5.2 ± 0.9°, 6.8 ± 1.1°, and 7.4 ± 1.0° in Groups 1, 2, and 3, respectively. Incisor protrusion occurred in Groups 1 and 2 (upper central incisor tip to maxillary vertical reference plane (mm) increase: 2.2 ± 0.5 mm and 1.5 ± 0.4 mm), while Group 3 demonstrated incisor retrusion (−0.8 ± 0.3 mm) (*P* < .01). Group 3 showed significantly less increase in overjet (0.4 ± 0.2 mm) compared to Groups 1 (2.5 ± 0.6 mm) and 2 (1.7 ± 0.5 mm) (*P* < .01).

**Conclusion:**

The double miniscrew-supported pendulum appliance demonstrated superior clinical efficiency with reduced treatment duration and minimal anchorage loss. This approach provided better control of incisor position during maxillary molar distalization compared to conventional and single miniscrew designs.

## 1. Introduction

Class II malocclusion is characterized by anterior positioning of the maxilla and/or maxillary dentition, posterior positioning of the mandible and/or mandibular dentition, or a combination of both.^[1]^ In skeletal Class 1, dental Class II cases, dental relationship can be treated by tooth extraction from maxillary arch or distalization on the maxillary arch.

In the literature, many techniques have been introduced for distalization, which is an alternative method to extraction in the upper jaw in the treatment of Dental Class II malocclusions. The advantages of extraoral appliances are that they are stable and have few side effects. However, the use of extraoral appliances in treatments has decreased today because of the requirement that patients cooperate with the treatment and the fact that they are unaesthetic. Many methods such as pendulums, distal jets, NiTi open coil springs, and 3D Bimetric Arches are used in intraoral distalization.^[2]^ The main problem with these appliances is the loss of anchorage. Skeletal anchorage has been used to minimize anchorage loss and reduce side effects.

The pendulum appliance, which does not require cooperation and is a fixed intraoral distalization appliance, was introduced by Hilgers.^[3]^ The biggest disadvantage of the appliance is the loss of anchorage.^[3]^ With the increase in the use of miniscrews, various studies have been carried out on palatal miniscrew-supported pendulum appliances.^[4–8]^

The aim of this study was to compare the effects of a miniscrew-supported pendulum and the conventional pendulum appliance on the skeletal and dentoalveolar structures in eliminating the anchorage losses encountered in intraoral molar distalization appliances in individuals with Dental Class II malocclusion. In this study, the effect of distalization on the molars as well as the effect of distalization on the incisors was investigated. In addition, the dental and skeletal effects of different pendulum designs were comparatively examined.

## 2. Materials and methods

### 2.1. Ethical approval and patient selection

Ethical approval for this prospective study was obtained from the Clinical Research Ethics Committee of the Faculty of Dentistry, Ankara University (Approval Number: 36290600/78). All individuals included in the study were informed about the treatment to be applied and an informed consent form was signed by the patients and their parents.

A total of 30 patients who presented to the Department of Orthodontics at Ankara University and received treatment with pendulum appliances were enrolled. Lateral cephalometric radiographs were obtained at 2 time points: before the initiation of molar distalization and immediately after achieving a bilateral Class I molar relationship.

### 2.2. Power analysis and grouping

A priori power analysis was performed using G*Power software (version 3.1; Heinrich-Heine-Universität Düsseldorf, Düsseldorf, Germany)^[9]^ to determine the required sample size. The analysis indicated that a minimum of 9 participants per group would be required to detect a statistically significant difference with 80% power at an alpha level of 0.05. With an effect size of f = 0.66, an α of 0.05, and a total sample size of 30 (10 participants per group), the study achieved a statistical power of 0.88. This analysis justified the chosen sample size for detecting significant differences using one-way analysis of variance. Therefore, 10 individuals were included in each group to ensure adequate power. The conventional pendulum appliance group was Group 1, the single miniscrew pendulum appliance group was Group 2, and the double miniscrew pendulum appliance group was Group 3. There were 10 individuals in each group. Age and gender distributions are shown in Table 2.

**Table 1:** Baseline demographic and clinical characteristics of the participants.

		Group				Kruskall–Wallis H test		
		Girl (N)	Boy (N)	Mean	SD	KWH	*P*	Comparison
Age	Group 1	8	2	13.6	1.2	2.6	.265	-
	Group 2	6	4	14.5	1.1			
	Group 3	7	3	14.3	1.2			
	Total	21	9	14.1	1.2			
Duration of treatment	Group 1	8	2	8.0	0.7	15.7	.0001****	1–2 1–3
	Group 2	6	4	7.3	0.5			
	Group 3	7	3	6.4	0.5			
	Total	21	9	7.2	0.9			

KWH = Kruskall–Wallis H test, N = number; SD = standard deviation.

**** *P* < .0001.

**Table 2:** Measurement reliability analysis using intraclass correlation coefficient (ICC).

	ICC	*P*
SNA (°)	1	.0001
SNB (°)	1	.0001
ANB (°)	0.98	.0001
SN/GoGN (°)	0.96	.0001
SN/PP (°)	0.95	.0001
A-VRP (mm)	0.98	.0001
A-MaxVRP (mm)	0.97	.0001
N-Me (mm)	0.965	.0001
ANS-Me (mm)	1	.0001
U1/MaxHRP (°)	0.98	.0001
U6/MaxHRP (°)	0.99	.0001
U7/MaxHRP (°)	0.92	.0001
Overjet (mm)	1	.0001
Overbite (mm)	1	.0001
U1t-MaxVRP (mm)	0.98	.0001
U6t-MaxVRP (mm)	0.96	.0001
U7t-MaxVRP (mm)	0.97	.0001
U1t-MaxHRP (mm)	1	.0001
U6t-MaxHRP (mm)	0.94	.0001
U7t-MaxHRP (mm)	0.98	.0001

A-MaxVRP = A point to maxillary vertical reference plane (mm), A-VRP = A point to vertical reference plane (mm), N-Me = nasion to menton (anterior facial height) (mm), SNA = sella–nasion–A point angle, SNB = sella–nasion–B point angle, SN/GoGn = mandibular plane (gonion–gnathion) to sella–nasion angle, SN/PP = sella–nasion to palatal plane angle, U1/MaxHRP = upper central incisor long-axis angle to Max.HRP (°), U1t-MaxHRP = upper central incisor tip to Max.HRP (mm), U1t-MaxVRP = upper central incisor tip to Max.VRP (mm), U6/MaxHRP (°) = upper first molar long-axis angle to Max.HRP (°), U6t-MaxHRP = upper first molar mesial cusp tip to Max.HRP (mm), U6t-MaxVRP = upper first molar mesial cusp tip to Max.VRP (mm), U7/MaxHRP (°) = upper second molar long-axis angle to Max.HRP (°), U7t-MaxHRP = upper second molar mesial cusp tip to Max.HRP (mm), U7t-MaxVRP = upper second molar mesial cusp tip to Max.VRP (mm).

### 2.3. Randomization and allocation concealment

**Figure 1.**
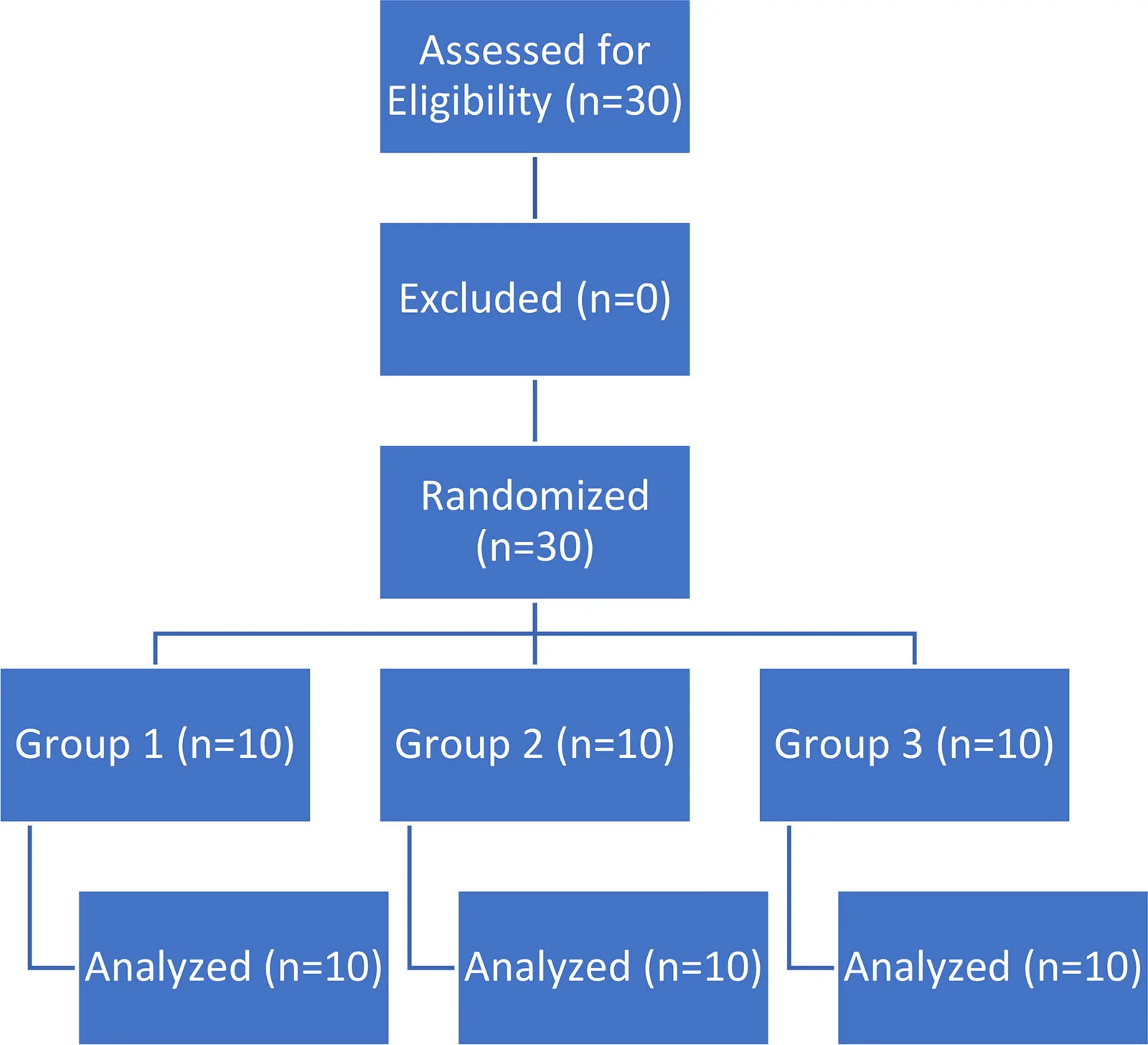
CONSORT flow diagram depicting participant enrollment, allocation, follow-up, and analysis.

The randomization process was carried out using a computer-generated random number sequence to assign patients impartially into 3 groups. Group assignments were concealed in sealed opaque envelopes and opened by an independent researcher who was not involved in treatment procedures or outcome assessment. This approach ensured proper allocation concealment and minimized the risk of selection bias, in accordance with Consolidated Standards of Reporting Trials guidelines.^[10]^ Figure 1 shows the Consolidated Standards of Reporting Trials flow diagram detailing the participant flow through each stage of the study.

### 2.4. Inclusion criteria

1. Skeletal Class I (0°< A point–nasion–B point angle [ANB] < 4°), dental full Angle Class II relationship.
2. Non-extraction treatment option.
3. Full eruption of maxillary second molars.
4. Patients with an overjet > 4 mm and/or maxillary anterior crowding >7 mm.
5. Good oral hygiene and no prior orthodontic treatment.

### 2.5. Appliance design and clinic procedure

In Group 1, the pendulum appliance was applied without skeletal anchorage. Anchorage was achieved through a Nance button and the first and second premolars. In Groups 2 and 3, self-drilling titanium miniscrews (2 mm in diameter, 10 mm in length; Biomaterials Korea, Seoul, Korea) were used to provide skeletal anchorage. A total of 0.5 mL of local anesthetic was administered at the insertion sites. Miniscrews were placed manually by the same surgeon (M.K.E). In Group 2, 1 miniscrew was inserted unilaterally to either the right or left side of the midpalatal suture. In Group 3, 2 miniscrews were placed symmetrically on both sides of the midpalatal suture, approximately 4 to 5 mm posterior to the incisive foramen in the paramedian region, achieving monocortical engagement. Pilot drilling was not required. To minimize postoperative infection risk, a dental extraction protocol was followed, and patients were advised to use analgesics and antiseptic mouthwash.

**Figure 2.**
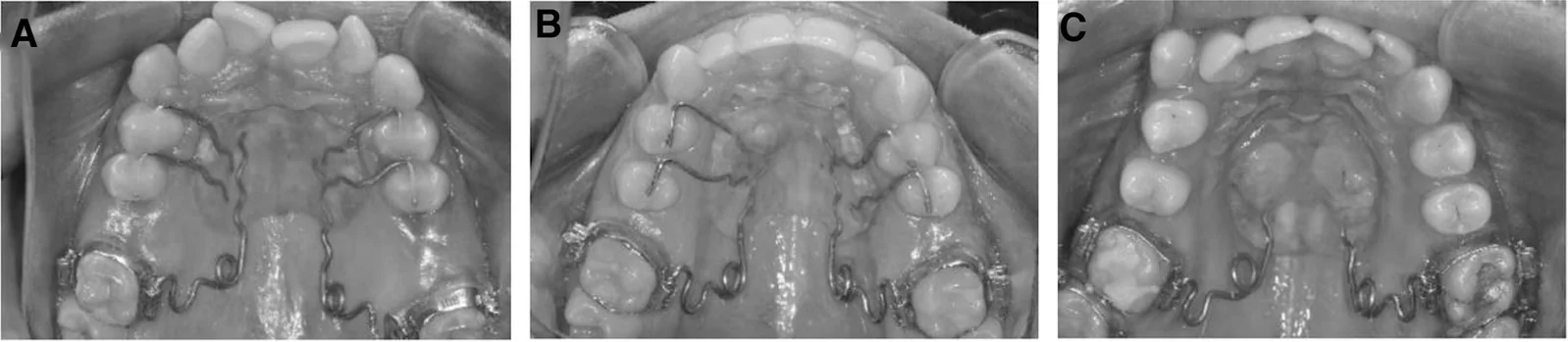
(A) Pendulum appliance; (B) single miniscrew-supported pendulum appliance; (C) double miniscrew-supported pendulum appliance.

After miniscrew placement, palatal sheathed molar bands were fitted to the first molars and alginate impressions were taken. The pendulum appliance was constructed using the Hilgers method.^[3]^ A Nance button was fabricated in all groups. In Groups 2 and 3, during laboratory construction, the heads of the miniscrews were blocked out on the stone model using wax. In Group 2, to prevent unwanted rotation of the acrylicbutton during activation, 0.9-inch full round stainless steel occlusal arms were bent and extended to the premolar teeth. In contrast, Group 3 relied solely on skeletal anchorage, and therefore no auxiliary extensions were applied to the premolars. The pendulum springs were bent from 0.036 inch titanium–molybdenum alloy (Beta Titanium) wire, incorporating a “U” loop to generate distalization force. Additionally, as recommended by Byloff et al,^[11]^ an uprighting bend was incorporated at the end of the titanium–molybdenum alloy wire to control molar tipping (Fig. 2). Once the wire framework was adapted and waxed on the model, the acrylic button was fabricated to cover the palatal vault, leaving recesses at the miniscrew sites. The screw–acrylic interface and occlusal arms were bonded intraorally using a flowable composite (Flowable Restorative, 3M ESPE, MN, USA).

The pendulum springs were activated at 90°, delivering approximately 250 g of distalizing force. Finally, activated springs were placed in the lingual sheaths on the first molar bands. The patients were checked every 4 weeks and the springs reactivated if necessary.

### 2.6. Radiographic evaluation

All lateral cephalometric radiographs were taken using the Sirona Orthophos XG Plus DS/Ceph device, which has a fixed magnification factor of 1.11 (11%). All radiographs were taken in centric occlusion, with the Frankfurt horizontal plane parallel to the ground. Standardized head positioning was maintained throughout image acquisition to ensure consistency. Cephalometric landmarks were digitally identified using CorelDRAW software (Corel Corporation, Ottawa, Ontario, Canada), and all linear and angular measurements were recorded accordingly.

To ensure measurement accuracy and minimize potential bias, the individual responsible for patient treatment and appliance placement was different from the examiner conducting the cephalometric evaluations. The evaluator was blinded to the group allocation of the patients, employing a single-blind study design. All measurements were carried out by the same trained researcher.

Intra-examiner reliability was assessed by re-measuring the cephalometric radiographs of 9 randomly selected individuals (3 from each group) 15 days after the initial evaluation. The intraclass correlation coefficient was calculated for each variable to confirm the consistency and reliability of repeated measurements. This methodological step was adopted to minimize potential measurement bias and ensure reproducibility.

**Figure 3.**
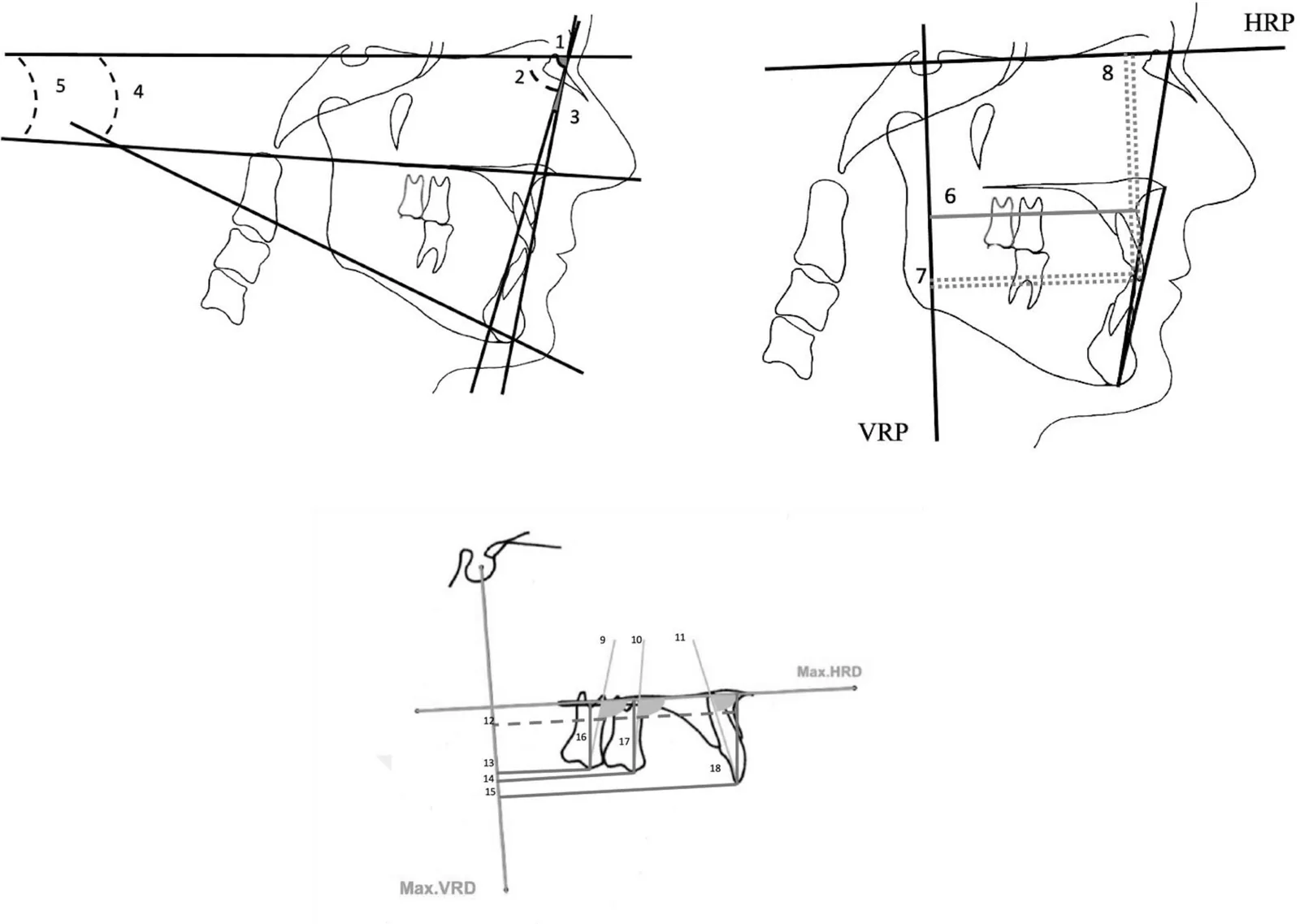
Cephalometric measurements: (1) SNA (°); (2) SNB (°); (3) ANB (°); (4) GoGn/SN (°); (5) SN/PP (°); (6) A-VRP (mm), distance from point A to the vertical reference plane; (7) overbite (mm); (8) overjet (mm); dental measurements: (9) U7/Max.HRP (°); (10) U6/Max.HRP (°); (11) U1/Max.HRP (°); (12) A-Max.HRP (mm); (13) U7–Max.VRP (mm); (14) U6–Max.VRP (mm); (15) U1–Max.VRP (mm); (16) U7–Max.HRP (mm); (17) U6–Max.HRP (mm); (18) U1–Max.HRP (mm). ANB = A point–nasion–B point angle, A-VRP = A point to vertical reference plane (mm), GoGn/SN = mandibular plane (gonion–gnathion) to sella–nasion angle, SN/PP = sella–nasion to palatal plane angle, SNA = sella–nasion–A point angle, SNB = sella–nasion–B point angle, U1/Max.HRP = upper central incisor long-axis angle to Max.HRP (°), U7 = maxillary second molar landmark/measurement, U7/MaxHRP = upper second molar long-axis angle to Max.HRP (°).

For reference planes, the SN line was taken as the horizontal reference plane (HRP), and a perpendicular line drawn from point S served as the vertical reference plane (VRP). When performing local measurements in the maxilla, the plane drawn between anterior nasal spine (ANS) and posterior nasal spine was used as the maxillary horizontal reference plane (Max.HRP), and the perpendicular line drawn from point S to this plane was taken as the maxillary vertical reference plane (Max.VRP). All reference points and measurement axes used in this study are shown in Figure 3.

Cephalometric radiographs were taken at 2 specific time points:

1. before the initiation of molar distalization, and
2. immediately after the achievement of a bilateral Class I molar relationship.

Since the post-treatment radiographs were scheduled according to this individualized clinical endpoint, the total treatment duration naturally varied across the 3 groups. The mean treatment durations were 8.0 ± 0.7 months in Group 1, 7.3 ± 0.5 months in Group 2, and 6.4 ± 0.5 months in Group 3, as presented in Table 2.

### 2.7. Statistical analysis

All statistical analyses were performed using IBM Statistical Package for the Social Sciences Statistics for Windows, Version 21.0 (Armonk: IBM Corp.). The Shapiro–Wilk test was used to assess the normality of data distribution for each variable. For normally distributed variables, descriptive statistics were expressed as mean ± standard deviation (SD) and comparisons were performed using 1-way analysis of variance. For variables not following a normal distribution, the nonparametric Kruskal–Wallis H test was applied, and results were reported as median and interquartile range where applicable.

For within-group comparisons, the paired samples *t* test was utilized. The significance level was set at α = 0.05, and a *P*-value less than .05 was considered statistically significant.

## 3. Results

No miniscrew failure (e.g., loosening or loss) was observed in any patient throughout the treatment. All miniscrews remained stable until the completion of the distalization phase.

To assess intra-examiner reliability, a total of 9 cephalometric radiographs, randomly selected from 3 patients in each group, were remeasured by the same examiner 15 days after the initial evaluation. Intraclass correlation coefficient values for all variables ranged between 0.92 and 1.00, indicating excellent measurement reproducibility (Table 1).

The demographic characteristics and treatment durations of the study groups are presented in Table 2. The treatment duration of Group 1 was found to be statistically significantly higher than that of the other groups. The mean ages were 13.6 ± 1.2 years in Group 1, 14.5 ± 1.1 years in Group 2, and 14.3 ± 1.2 years in Group 3. According to the Kruskal–Wallis H test, there was no statistically significant difference in mean age among the groups (*P* > .05). Treatment duration was significantly longer in Group 1 compared to the other groups (*P* < .05).

There were no statistically significant differences in baseline cephalometric measurements among the 3 groups (*P* > .05), confirming the homogeneity of initial conditions.

In Group 1, statistically significant increases were observed in the ANB angle, mandibular plane (gonion–gnathion) to sella–nasion angle, and sella–nasion to palatal plane angle (*P* < .05). Nasion to menton (anterior facial height) (mm) (N-Me) and anterior nasal spine to menton (lower anterior facial height) (mm) (ANS-Me) distances also showed significant increases (*P* < .0001). The maxillary central incisor landmark/measurement (U1)/MaxHRP angle decreased significantly, while the maxillary first molar landmark/measurement (U6)/MaxHRP and upper second molar long-axis angle to Max.HRP (°) (U7/MaxHRP) angles increased (*P* < .05). Overjet increased and overbite decreased significantly. Upper central incisor tip to Max.VRP (mm) (U1t-MaxVRP) and upper first molar mesial cusp tip to Max.HRP (mm) distances increased (*P* < .05), whereas upper first molar mesial cusp tip to Max.VRP (mm) (U6t-MaxVRP) and upper second molar mesial cusp tip to Max.VRP (mm) (U7t-MaxVRP) distances decreased significantly (*P* < .0001) (Table 3).

**Table 3:** Evaluation of the differences between the mean values of the conventional pendulum group (Group 1) before treatment and after distalization with paired *t* test.

	T1		T2		T1–T2		*P*
	Mean	SD	Mean	SD	Mean	SD	
Skeletal measurements							
SNA (°)	79.71	3.07	79.97	3.33	.26	1.52	.607
SNB (°)	77.38	2.95	76.77	3.11	-.61	1.25	.156
ANB (°)	2.33	1.46	3.20	1.55	.87	1.12	.036*
SN/GoGN (°)	30.42	4.12	32.40	5.01	1.98	2.06	.014*
SN/PP (°)	7.76	2.73	8.58	2.96	.81	.97	.027*
A-VRP (mm)	67.24	5.89	65.75	5.77	-1.49	2.35	.075
A-MaxVRP (mm)	75.99	5.37	75.29	5.17	-.70	2.19	.336
N-Me (mm)	132.89	6.63	136.41	6.34	3.52	1.68	.0001****
ANS-Me (mm)	73.32	6.19	76.41	5.89	3.09	1.32	.0001****
Dental measurements							
U1/MaxHRP (°)	68.01	6.19	62.56	4.80	-5.45	3.76	.001***
U6/MaxHRP (°)	101.62	5.65	113.58	6.00	11.96	4.77	.0001****
U7/MaxHRP (°)	108.04	6.29	119.907	6.531	11.87	5.25	.0001****
Overjet (mm)	5.79	3.04	7.95	3.29	2.16	1.09	.0001****
Overbite (mm)	4.82	3.00	3.00	2.17	-1.83	1.07	.0001****
U1t-MaxVRP (mm)	79.51	5.85	81.57	3.01	2.05	4.36	.041*
U6t-MaxVRP (mm)	45.93	5.57	40.03	5.21	-5.9	1.64	.0001****
U7t-MaxVRP (mm)	28.94	8.76	25.29	8.20	-3.65	1.65	.0001****
U1t-MaxHRP (mm)	31.70	4.63	31.30	4.44	-0.4	1.41	.399
U6t-MaxHRP (mm)	24.50	3.18	25.51	2.45	1.01	1.41	.049*
U7t-MaxHRP (mm)	18.73	5.18	18.79	2.79	0.05	3.54	.963

A-VRP = A point to vertical reference plane (mm), N-Me = nasion to menton (anterior facial height) (mm), SD = standard deviation, SNA = sella–nasion–A point angle, SNB = sella–nasion–B point angle, SN/GoGn = mandibular plane (gonion–gnathion) to sella–nasion angle, SN/PP = sella–nasion to palatal plane angle, U1/MaxHRP = upper central incisor long-axis angle to Max.HRP (°), U1t-MaxHRP = upper central incisor tip to Max.HRP (mm), U1t-MaxVRP = upper central incisor tip to Max.VRP (mm), U6/MaxHRP (°) = upper first molar long-axis angle to Max.HRP (°), U6t-MaxHRP = upper first molar mesial cusp tip to Max.HRP (mm), U6t-MaxVRP = upper first molar mesial cusp tip to Max.VRP (mm), U7/MaxHRP (°) = upper second molar long-axis angle to Max.HRP (°), U7t-MaxHRP = upper second molar mesial cusp tip to Max.HRP (mm), U7t-MaxVRP = upper second molar mesial cusp tip to Max.VRP (mm).

* *P*<.05.

*** *P*<.001.

**** *P*<.0001.

In Group 2, a significant decrease in sella–nasion–B point angle (SNB) angle (*P* < .01) and a significant increase in ANB angle (*P* < .05) were observed. N-Me and ANS-Me values also significantly increased (*P* < .01 and *P* < .0001, respectively). The upper central incisor long-axis angle to Max.HRP (°) (U1/MaxHRP angle) decreased significantly (*P* < .05), while upper first molar long-axis angle to Max.HRP (°) increased (*P* < .0001) and U7/MaxHRP did not show significant change. Overjet increased significantly. U1t-MaxVRP and upper first molar mesial cusp tip to Max.HRP (mm) increased (*P* < .05), while U6t-MaxVRP and U7t-MaxVRP decreased significantly (*P* < .0001) (Table 4).

**Table 4:** Evaluation of the differences between the mean values of the single miniscrew-supported pendulum group (Group 2) before treatment and after distalization with paired *t* test.

	T1		T2		T1–T2		*P*
	Mean	SD	Mean	SD	Mean	SD	
Skeletal measurements							
SNA (°)	78.86	4.00	79.10	3.70	.24	1.07	.494
SNB (°)	76.19	3.34	75.34	3.23	-.86	.66	.003**
ANB (°)	2.67	0.77	3.76	0.94	1.10	1.07	.011*
SN/GoGN (°)	31.83	5.62	33.30	5.95	1.47	2.80	.131
SN/PP (°)	7.23	3.59	7.55	2.76	.32	1.13	.401
A-VRP (mm)	68.84	3.15	68.44	4.14	-.40	2.32	.596
A-MaxVRP (mm)	76.44	5.22	76.87	4.70	.43	1.13	.263
N-Me (mm)	136.06	3.71	141.44	4.17	5.38	4.55	.005**
ANS-Me (mm)	77.09	5.00	82.02	4.21	4.93	2.13	.0001****
Dental measurements							
U1/MaxHRP (°)	65.83	10.52	63.99	11.29	-1.84	2.40	.038*
U6/MaxHRP (°)	97.23	5.99	110.49	6.95	13.26	4.53	.0001****
U7/MaxHRP (°)	95.81	30.00	109.159	5.899	13.35	6.13	.039*
Overjet (mm)	7.19	3.03	9.29	3.87	2.1	2.24	.016*
Overbite (mm)	5.91	1.83	3.88	2.06	-2.03	1.12	.0001****
U1t-MaxVRP (mm)	78.25	10.64	80.27	11.29	2.02	2.2	.009**
U6t-MaxVRP (mm)	41.13	9.29	34.57	8.13	-6.56	3.6	.0001****
U7t-MaxVRP (mm)	34.30	4.41	28.99	4.63	-5.31	2.39	.0001****
U1t-MaxHRP (mm)	33.09	2.24	33.22	2.36	0.13	0.69	.559
U6t-MaxHRP (mm)	24.80	1.78	25.30	4.39	0.5	4.02	.703
U7t-MaxHRP (mm)	21.34	1.81	20.53	2.86	-0.48	2.57	.243

A-VRP = A point to vertical reference plane (mm), N-Me = nasion to menton (anterior facial height) (mm), SD = standard deviation, SNA = sella–nasion–A point angle, SNB = sella–nasion–B point angle, SN/GoGn = mandibular plane (gonion–gnathion) to sella–nasion angle, SN/PP = sella–nasion to palatal plane angle, U1/MaxHRP = upper central incisor long-axis angle to Max.HRP (°), U1t-MaxHRP = upper central incisor tip to Max.HRP (mm), U1t-MaxVRP = upper central incisor tip to Max.VRP (mm), U6/MaxHRP (°) = upper first molar long-axis angle to Max.HRP (°), U6t-MaxHRP = upper first molar mesial cusp tip to Max.HRP (mm), U6t-MaxVRP = upper first molar mesial cusp tip to Max.VRP (mm), U7/MaxHRP (°) = upper second molar long-axis angle to Max.HRP (°), U7t-MaxHRP = upper second molar mesial cusp tip to Max.HRP (mm), U7t-MaxVRP = upper second molar mesial cusp tip to Max.VRP (mm).

* *P* < .05.

** *P* < .01.

**** *P* < .0001.

In Group 3, a significant decrease in SNB angle and a corresponding significant increase in ANB angle were found (*P* < .01). While N-Me and ANS-Me values increased, the change was not statistically significant. Unlike Groups 1 and 2, the U1/MaxHRP angle increased in Group 3, indicating incisor retrusion. The upper first molar long-axis angle to Max.HRP (°) and U7/MaxHRP angles increased, while overjet showed the least increase among all groups. U1t-MaxVRP remained stable, while U6t-MaxVRP and U7t-MaxVRP decreased significantly (*P* < .0001) (Table 5).

**Table 5:** Evaluation of the differences between the mean values of the double miniscrew-supported pendulum group (Group 3) before treatment and after distalization with paired *t* test.

	T1		T2		T1–T2		*P*
	Mean	SD	Mean	SD	Mean	SD	
Skeletal measurements							
SNA (°)	81.82	2.39	81.99	2.20	.17	.73	.477
SNB (°)	78.70	2.41	76.26	1.96	-2.44	1.27	.0001****
ANB (°)	3.11	1.06	5.72	1.34	2.61	1.02	.0001****
SN/GoGN (°)	32.60	4.36	36.28	4.54	3.68	3.10	.005**
SN/PP (°)	6.42	2.32	7.66	3.23	1.25	3.92	.341
A-VRP (mm)	64.81	6.91	65.79	7.24	.98	1.81	.121
A-MaxVRP (mm)	72.49	8.86	73.89	8.84	1.40	2.04	.158
N-Me (mm)	130.46	6.09	134.51	3.86	4.05	5.85	.056
ANS-Me (mm)	74.34	6.67	79.00	5.26	4.66	3.13	.001***
Dental measurements							
U1/MaxHRP (°)	67.21	2.07	67.62	2.13	.41	.65	.039*
U6/MaxHRP (°)	97.68	6.90	111.91	6.42	14.23	7.19	.0001****
U7/MaxHRP (°)	106.55	5.66	121.28	8.53	14.73	3.17	.041*
Overjet (mm)	4.74	1.13	5.04	1.03	0.31	0.78	.245
Overbite (mm)	3.92	1.34	2.32	1.51	-1.6	0.91	.0001****
U1t-MaxVRP (mm)	80.61	6.10	80.66	5.98	0.05	0.35	.656
U6t-MaxVRP (mm)	46.65	3.64	39.68	3.21	-6.97	2.84	.0001****
U7t-MaxVRP (mm)	35.04	3.88	28.46	3.95	-6.58	2.25	.0001****
U1t-MaxHRP (mm)	33.43	2.37	33.66	2.45	0.23	0.46	.151
U6t-MaxHRP (mm)	25.53	1.87	26.08	2.23	0.56	1.1	.143
U7t-MaxHRP (mm)	21.47	1.03	21.00	2.66	-0.81	2.04	.571

A-VRP = A point to vertical reference plane (mm), N-Me = nasion to menton (anterior facial height) (mm), SD = standard deviation, SNA = sella–nasion–A point angle, SNB = sella–nasion–B point angle, SN/GoGn = mandibular plane (gonion–gnathion) to sella–nasion angle, SN/PP = sella–nasion to palatal plane angle, U1/MaxHRP = upper central incisor long-axis angle to Max.HRP (°), U1t-MaxHRP = upper central incisor tip to Max.HRP (mm), U1t-MaxVRP = upper central incisor tip to Max.VRP (mm), U6/MaxHRP (°) = upper first molar long-axis angle to Max.HRP (°), U6t-MaxHRP = upper first molar mesial cusp tip to Max.HRP (mm), U6t-MaxVRP = upper first molar mesial cusp tip to Max.VRP (mm), U7/MaxHRP (°) = upper second molar long-axis angle to Max.HRP (°), U7t-MaxHRP = upper second molar mesial cusp tip to Max.HRP (mm), U7t-MaxVRP = upper second molar mesial cusp tip to Max.VRP (mm).

* *P*<.05.

** *P*<.01.

*** *P*<.001.

**** *P*<.0001.

When comparing the groups (Table 6), statistically significant differences were found in SNB, ANB, A point to vertical reference plane (mm), A point to maxillary vertical reference plane (mm), U1/MaxHRP, U1t-MaxVRP, U6t-MaxVRP, U7t-MaxVRP, and overjet values. Pairwise comparisons revealed that Group 3 showed significantly greater distal movement with less incisor protrusion compared to Groups 1 and 2 (*P* < .05). The most favorable outcomes in terms of treatment duration and dentoalveolar side effects were observed in Group 3.

**Table 6:** Comparison of the differences between the mean values of the measurements at the beginning and the end of the treatment with the Kruskall–Wallis *H* test between groups.

		Group			Kruskall–Wallis *H* test		
		N	Mean	SD	KWH	*P*	Comparison
Skeletal measurements							
SNA (°) difference	Group 1	10	.26	1.52	0	.981	–
	Group 2	10	.24	1.07			
	Group 3	10	.17	.73			
	Total	30	.22	1.12			
SNB (°) difference	Group 1	10	-.61	1.25	10.7	.005**	3–1 3–2
	Group 2	10	-.86	.66			
	Group 3	10	-2.44	1.27			
	Total	30	-1.30	1.34			
ANB (°) difference	Group 1	10	.87	1.12	11.8	.003**	3–1 3–2
	Group 2	10	1.10	1.07			
	Group 3	10	2.61	1.02			
	Total	30	1.53	1.30			
SN/GoGN (°) difference	Group 1	10	1.98	2.06	1.9	.381	–
	Group 2	10	1.47	2.80			
	Group 3	10	3.68	3.10			
	Total	30	2.37	2.76			
SN/PP (°) difference	Group 1	10	.81	.97	0.9	.647	–
	Group 2	10	.32	1.13			
	Group 3	10	1.25	3.92			
	Total	30	.79	2.37			
A-VRP (mm) difference	Group 1	10	-1.49	2.35	6.6	.037*	1–2 1–3
	Group 2	10	-.40	2.32			
	Group 3	10	.98	1.81			
	Total	30	-.31	2.34			
A-MaxVRP(mm) difference	Group 1	10	-.70	2.19	7.9	.019*	1–2 1–3
	Group 2	10	.43	1.13			
	Group 3	10	1.40	2.04			
	Total	30	.37	1.98			
N-Me (mm) difference	Group 1	10	3.52	1.68	1	.618	–
	Group 2	10	5.38	4.55			
	Group 3	10	4.05	5.85			
	Total	30	4.32	4.31			
ANS-Me (mm) difference	Group 1	10	3.09	1.32	3.4	.186	–
	Group 2	10	4.93	2.13			
	Group 3	10	4.66	3.13			
	Total	30	4.23	2.38			
Dental measurements							
U1/MaxHRP (°) difference	Group 1	10	-5.45	3.76	15.9	.0001****	1–2 1–3 2–3
	Group 2	10	-1.84	2.40			
	Group 3	10	.41	.65			
	Total	30	-2.30	3.51			
U6/MaxHRP (°) difference	Group 1	10	11.96	4.77	0.7	.716	–
	Group 2	10	13.26	4.53			
	Group 3	10	14.23	7.19			
	Total	30	13.15	5.51			
U7/MaxHRP (°) difference	Group 1	10	11.87	5.25	2.3	.312	–
	Group 2	10	13.35	6.13			
	Group 3	10	14.73	3.17			
	Total	30	13.32	4.97			
Overjet (mm) difference	Group 1	10	2.16	1.09	12.2	.002**	3–1 3–2
	Group 2	10	2.1	2.24			
	Group 3	10	0.31	0.78			
	Total	30	1.52	1.7			
Overbite (mm) difference	Group 1	10	-1.83	1.07	1.5	.468	–
	Group 2	10	-2.03	1.12			
	Group 3	10	-1.6	0.91			
	Total	30	-1.82	1.02			
U1t-MaxVRP (mm) difference	Group 1	10	2.05	4.36	11.3	.003**	3–1 3–2
	Group 2	10	2.02	2.2			
	Group 3	10	0.05	0.35			
	Total	30	1.48	2.91			
U6t-MaxVRP (mm) difference	Group 1	10	-5.9	1.64	1.5	.046*	3–1 3–2
	Group 2	10	-6.56	3.6			
	Group 3	10	-6.97	2.84			
	Total	30	-6.48	2.75			
U7t-MaxVRP (mm) difference	Group 1	10	-3.65	1.65	9.7	.008**	3–1 3–2
	Group 2	10	-5.31	2.39			
	Group 3	10	-6.58	2.25			
	Total	30	-5.18	2.38			
U1t-MaxHRP (mm) difference	Group 1	10	-0.4	1.41	1.4	.507	–
	Group 2	10	0.13	0.69			
	Group 3	10	0.23	0.46			
	Total	30	-0.01	0.96			
U6t-MaxHRP (mm) difference	Group 1	10	1.01	1.41	2.2	.331	–
	Group 2	10	0.5	4.02			
	Group 3	10	0.56	1.1			
	Total	30	0.69	2.46			
U7t-MaxHRP (mm) difference	Group 1	10	0.05	3.54	0.1	.951	–
	Group 2	10	-0.48	2.57			
	Group 3	10	-0.81	2.04			
	Total	30	-0.41	2.71			

A-VRP = A point to vertical reference plane (mm), KWH = Kruskall–Wallis *H* test, N = number, N-Me = nasion to menton (anterior facial height) (mm), SD = standard deviation, SNA = sella–nasion–A point angle, SNB = sella–nasion–B point angle, SN/GoGn = mandibular plane (gonion–gnathion) to sella–nasion angle, SN/PP = sella–nasion to palatal plane angle, U1/MaxHRP = upper central incisor long-axis angle to Max.HRP (°), U1t-MaxHRP = upper central incisor tip to Max.HRP (mm), U1t-MaxVRP = upper central incisor tip to Max.VRP (mm), U6/MaxHRP (°) = upper first molar long-axis angle to Max.HRP (°), U6t-MaxHRP = upper first molar mesial cusp tip to Max.HRP (mm), U6t-MaxVRP = upper first molar mesial cusp tip to Max.VRP (mm), U7/MaxHRP (°) = upper second molar long-axis angle to Max.HRP (°), U7t-MaxHRP = upper second molar mesial cusp tip to Max.HRP (mm), U7t-MaxVRP = upper second molar mesial cusp tip to Max.VRP (mm).

* *P*<.05.

** *P*<.01.

**** *P*<.0001.

## 4. Discussion

Molar distalization has been considered a viable alternative to tooth extraction in the management of Class II malocclusions, offering the benefit of achieving a Class I molar relationship and providing space to alleviate anterior crowding.^[12]^

Although extraoral appliances have traditionally been employed for molar distalization, their primary limitation lies in the requirement for patient cooperation. In response to this limitation, a variety of intraoral distalization devices (such as pendulums, distal jets, and the Jones Jig) have been developed.^[2]^ However, some intraoral devices like the 3D Bimetric Maxillary Distalization Arch and superelastic NiTi wire systems still depend on Class II elastics, and therefore, on patient compliance.^[13]^

In open coil springs, pendulums, and distal jet devices, which eliminate the need for cooperation, anchorage loss is a major problem because support is taken from the maxillary anterior region. Due to the force applied, mesial movement and tipping can be seen in the teeth that are supported, and this causes an increase in protrusion and overjet in the incisors.

Miniscrews, in particular, have been used to achieve more effective distalization and to eliminate side effects due to anchorage loss.^[5,6]^ There is no previous studies investigated the effects of 2 types of miniscrew-supported pendulum appliance (1 and 2 miniscrews) and pendulum appliance with conventional anchorage on dentoalveolar structures were compared in individuals.

In our study, a single miniscrew in Group 2 was inserted unilaterally (either right or left) to the paramedian region of the midpalatal suture, whereas in Group 3, 2 miniscrews were placed symmetrically on both sides of the suture. Additionally, auxiliary arms extended toward the premolars were incorporated only in Groups 1 and 2. Group 3 relied solely on skeletal anchorage.

Activation of springs changes the applied force in intraoral distalization appliances with a pendulum. In studies, the activation of the springs has been between 45° and 90°.^[3,4]^ The activation of the springs in our study was determined as 90° and a force of 250 g was applied in all groups.

The center of resistance of the upper first molars is around the furcation region of the tooth, and the distalization forces are usually applied below the center of resistance, thus distal tipping becomes unavoidable. It has been reported that less tipping is observed because the distalization force applied from the palatal region, a region closer to the resistance center of the first molar tooth.^[14]^

Some researchers have mentioned a backward movement at the A point due to the protrusion of the incisors as a result of distalization.^[15]^ Also, researchers have reported that the distalization force is due to the remodeling that occurs as a result of the root movement caused by the protrusion seen in the incisors as a side effect and that the A point moves forward.^[16,17]^ Researchers who said that point A remained constant stated that no change was observed at point A due to the short duration of the force.^[7,15]^

In most of the studies using miniscrews during distalization, no movement was observed at A point, while a forward movement was observed at point A in some studies.^[18]^ In our study sella–nasion–A point angle did not show a statistically significant change in any group. A point to vertical reference plane (mm) and A-Max.VRP were not change significantly in any group, but the decrease in Group 1 was statistically significant compared to the other groups.

In some studies, a decrease in SNB° was observed, as in our study.^[6,19]^ The increase in the ANB° was higher in Group 3 compared to the other groups. It is thought that the increase in the ANB° is associated with the decrease in the SNB° and the increase in the mandibular plane (gonion–gnathion) to sella–nasion angle (GoGn/SN°). GoGn/SN° increased in all groups, as in other studies, but the increase was not statistically significant between the groups. There are also studies in the literature stating that there is no significant change in GoGn/SN° as a result of distalization.^[20–22]^

There are studies stating that the molar moves parallel with distalization,^[16,23]^ and that indicate that distal tipping is observed during distalization even when a miniscrew is used.^[5,15,17]^ In our study, distal movement with tipping of first molar was found statistically significant in all groups. Because of the premolars are not supported, all of the distalization force is transmitted to the molar, so distal tipping and movement are greater in Group 3.^[6,7]^ Distal tipping was observed in the first and second molars in all groups. The amount of distalization increased from Group 1 to Group 3, and distal tipping also increased from Group 1 to Group 3.

It has been reported in the literature, that distal tipping is also seen with distal movement during distalization in the upper second molars.^[15,17,22]^ In this study, distal tipping in the second molars was statistically significant.

There are studies in the literature suggesting that there is no change in the vertical measurements of the molar teeth,^[4,24]^ extrusion,^[16]^ and intrusion.^[25]^ Although extrusion of first molar was observed in all groups in the current study, it was not found to be statistically significant.

In conventional distalization appliances, the reciprocal force generated by the force applied to the molars causes 1.9° to 10° protrusion of the incisors.^[15,26]^ When miniscrew-supported distalization methods are used, retrusion^[5,7,23,25]^ or protrusion^[27]^ can be seen in incisors, while some researchers have reported no change.^[6,8]^

In this study, while protrusion was seen in Group 1 and Group 2, retrusion was seen in Group 3. This difference between the groups statistically significant. Similar with many studies, significantly more protrusion was observed in Group 1 compared to both groups.^[3,7,15,28]^

Previous studies have reported that the low amount of incisor retroclination in the skeletal anchorage supported pendulum is due to the short duration of treatment.^[5–7]^ In our study, we also observed a decrease in retroclination due to the short duration of treatment.

The overjet increased statistically significantly in Groups 1 and 2, but the increase was not found to be significant in Group 3. When the changes in the amount of overjet were compared between the groups, the differences were found to be statistically different.

In our study, the auxiliary arm was not extended to the premolars in Group 3. When the premolars are not included in the appliance, spontaneous distalization can occur in the premolar teeth through the transseptal fibers.^[4–6]^ Accordingly, spontaneous improvement in anterior crowding and reduction of overjet resulted in a shortening of the total treatment time.

From a clinical perspective, the use of self-drilling miniscrews with long necks provided excellent stability throughout the treatment period. No complications such as loosening, loss of screws, soft tissue irritation, inflammation, or infection were observed. This supports the reliability of skeletal anchorage systems in distalization procedures when appropriately planned.

## 5. Conclusion

In Group 1, anchorage loss led to significant incisor protrusion, whereas in Group 3, no anchorage loss was observed and statistically significant incisor retrusion occurred. Vertical measurements increased in all groups, particularly in the lower anterior facial height. Overjet increased in Groups 1 and 2, while it remained stable in Group 3. Overbite decreased across all groups. Group 3 demonstrated the shortest total treatment duration, which may be attributed to spontaneous alignment of the anterior crowding. These findings suggest that using 2 palatal miniscrews for skeletal anchorage offers better control over anterior dental movement and may contribute to a more efficient and stable distalization protocol.

## Author contributions

**Conceptualization:** Ayca Aksoy.

**Data curation:** Oyku Ozen.

**Formal analysis:** Oyku Ozen.

**Investigation:** Ayca Aksoy, Murat Kaan Erdem.

**Methodology:** Ayca Aksoy, Murat Kaan Erdem.

**Project administration:** Ayca Aksoy.

**Resources:** Ayca Aksoy.

**Software:** Oyku Ozen.

**Supervision:** Murat Kaan Erdem.

**Validation:** Oyku Ozen.

**Visualization:** Oyku Ozen.

**Writing – original draft:** Oyku Ozen, Murat Kaan Erdem.

**Writing – review & editing:** Ayca Aksoy, Oyku Ozen, Murat Kaan Erdem.

## References

[1] Ülgen M. Ortodontik Tedavi Prensipleri. Ankara: Ankara Üniversitesi Basimevi; 1983:161–96.

[2] Melsen B Verna C. A rational approach to orthodontic anchorage. Prog Orthod. 2000;1:10–22.

[3] Hilgers JJ. The pendulum appliance for Class II non-compliance therapy. J Clin Orthod. 1992;26:706–14. 1298751

[4] Kircelli BH Pektas ZO Kircelli C. Maxillary molar distalization with a bone-anchored pendulum appliance. Angle Orthod. 2006;76:650–9. 10.1043/0003-3219(2006)076[0650:MMDWAB]2.0.CO;2 16808573

[5] Escobar SA Tellez PA Moncada CA Villegas CA Latorre CM Oberti G. Distalization of maxillary molars with the bone-supported pendulum: a clinical study. Am J Orthod Dentofacial Orthop. 2007;131:545–9. 10.1016/j.ajodo.2006.08.012 17418723

[6] Oncag G Seckin O Dincer B Arikan F. Osseointegrated implants with pendulum springs for maxillary molar distalization: a cephalometric study. Am J Orthod Dentofacial Orthop. 2007;131:16–26. 10.1016/j.ajodo.2005.02.034 17208102

[7] Polat-Ozsoy O Kircelli BH Arman-Ozcirpici A Pektas ZO Uckan S. Pendulum appliances with 2 anchorage designs: conventional anchorage vs bone anchorage. Am J Orthod Dentofacial Orthop. 2008;133:339.e9–17. 10.1016/j.ajodo.2007.10.002 18331928

[8] Kaya B Sar C Arman-Ozcirpici A Polat-Ozsoy O. Palatal implant versus zygoma plate anchorage for distalization of maxillary posterior teeth. Eur J Orthod. 2013;35:507–14. 10.1093/ejo/cjs059 22968670

[9] Faul F Erdfelder E Buchner A Lang A-G. Statistical power analyses using G*Power 3.1: tests for correlation and regression analyses. Behav Res Methods. 2009;41:1149–60. 10.3758/BRM.41.4.1149 19897823

[10] Schulz KF Altman DG Moher D. CONSORT 2010 statement: updated guidelines for reporting parallel group randomized trials. BMJ. 2010;340:c332. 10.1097/AOG.0b013e3181d9d421 20410783

[11] Byloff FK Darendeliler MA Clar E Darendeliler A. Distal molar movement using the pendulum appliance. Part 2: the effects of maxillary molar root uprighting bends. Angle Orthod. 1997;67:261–70. 10.1043/0003-3219(1997)067<0261:DMMUTP>2.3.CO;2 9267574

[12] Luppanapornlarp S Johnston LE. The effects of premolar-extraction: a long-term comparison of outcomes in extraction and nonextraction Class II patients. Angle Orthod. 1993;63:257–72. 10.1043/0003-3219(1993)063<0257:TEOPAL>2.0.CO;2 8297050

[13] Locatelli R Bednar J Dietz VS Gianelly AA. Molar distalization with superelastic NiTi wire. J Clin Orthod. 1992;26:277–9. 1430176

[14] Antonarakis GS Kiliaridis S. Maxillary molar distalization with noncompliance intramaxillary appliances in Class II malocclusion: a systematic review. Angle Orthod. 2008;78:1133–40. 10.2319/101507-406.1 18947282

[15] Bondemark L Kurol J. Distalization of maxillary first and second molars simultaneously with repelling magnets. Eur J Orthod. 1992;14:264–72. 10.1093/ejo/14.4.264 1516658

[16] Keles A Sayinsu K. A new approach in maxillary molar distalization: intraoral bodily molar distalizer. Am J Orthod Dentofacial Orthop. 2000;117:39–48. 10.1016/s0889-5406(00)70246-0 10629518

[17] Fortini A Lupoli M Giuntoli F Franchi L. Dentoskeletal effects induced by rapid molar distalization with the first class appliance. Am J Orthod Dentofacial Orthop. 2004;125:697–704; discussion 704. 10.1016/j.ajodo.2003.06.006 15179394

[18] Keles A Erverdi N Sezen S. Bodily distalization of molars with absolute anchorage. Angle Orthod. 2003;73:471–82. 10.1043/0003-3219(2003)073<0471:BDOMWA>2.0.CO;2 12940570

[19] Bussick TJ McNamara JA. Dentoalveolar and skeletal changes associated with the pendulum appliance. Am J Orthod Dentofacial Orthop. 2000;117:333–43. 10.1016/s0889-5406(00)70238-1 10715093

[20] Joseph AA Butchart CJ. An evaluation of the pendulum distalizing appliance. Semin Orthod. 2000;6:129–35.

[21] Patel MP Janson G Henriques JFC. Comparative distalization effects of Jones jig and pendulum appliances. Am J Orthod Dentofacial Orthop. 2009;135:336–42. 10.1016/j.ajodo.2007.01.035 19268832

[22] Park HS Kwon TG Sung JH. Nonextraction treatment with microscrew implants. Angle Orthod. 2004;74:539–49. 10.1043/0003-3219(2004)074<0539:NTWMI>2.0.CO;2 15387034

[23] Papadopoulos MA. Orthodontic treatment of Class II malocclusion with miniscrew implants. Am J Orthod Dentofacial Orthop. 2008;134:604.e1–16; discussion 604. 10.1016/j.ajodo.2008.03.013 18984391

[24] Ghosh J Nanda RS. Evaluation of an intraoral maxillary molar distalization technique. Am J Orthod Dentofacial Orthop. 1996;110:639–46. 10.1016/s0889-5406(96)80041-2 8972811

[25] Lim SM Hong RK. Distal movement of maxillary molars using a lever-arm and mini-implant system. Angle Orthod. 2008;78:167–75. 10.2319/102506-438 18193963

[26] Muse DS Fillman MJ Emmerson WJ Mitchell RD. Molar and incisor changes with Wilson rapid molar distalization. Am J Orthod Dentofacial Orthop. 1993;104:556–65. 10.1016/s0889-5406(05)80439-1 8249931

[27] Kinzinger GSM Gulden N Yildizhan F Diedrich PR. Efficiency of a skeletonized distal jet appliance supported by miniscrew anchorage for noncompliance maxillary molar distalization. Am J Orthod Dentofacial Orthop. 2009;136:578–86. 10.1016/j.ajodo.2007.10.049 19815162

[28] Bozkaya E Tortop T Yuksel S Kaygisiz E. Evaluation of the effects of the hybrid Pendulum in comparison with the conventional Pendulum appliance. Angle Orthod. 2020;90:194–201. 10.2319/051719-340.1 PMC8051238 31642688

